# Walking between academia and industry to find successful solutions to biomedical challenges: an interview with Geoffrey Smith

**DOI:** 10.1242/dmm.022681

**Published:** 2015-10-01

**Affiliations:** Geoff is currently the Managing Director of Mars Ventures and a Managing Partner at Ascent Biomedical Ventures

## Abstract

Geoffrey W. Smith is currently the Managing Director of Mars Ventures. He actually started his studies with a Bachelor of Arts degree and a Doctorate in Law but then, in part by chance and in part by following in his family footsteps, he stepped into the healthcare and biotech field. Since then, he has successfully contributed to the birth of a number of healthcare companies and has also held academic positions at the Icahn School of Medicine at Mount Sinai and at The Rockefeller University in New York, teaching about the interface between science and business. During 2014 he served as Senior Editor on *Disease Models & Mechanisms*, bringing to the editorial team his valuable experience in drug development and discovery. In this interview, Geoff talks to Ross Cagan, Editor-in-Chief of *Disease Models & Mechanisms*, about how he developed his incredibly varied career, sharing his views about industry, academia and science publishing, and discussing how academia and industry can fruitfully meet to advance bioscience, train the scientists and stakeholders of the future, and drive the successful discovery of new therapeutics to treat human disease.


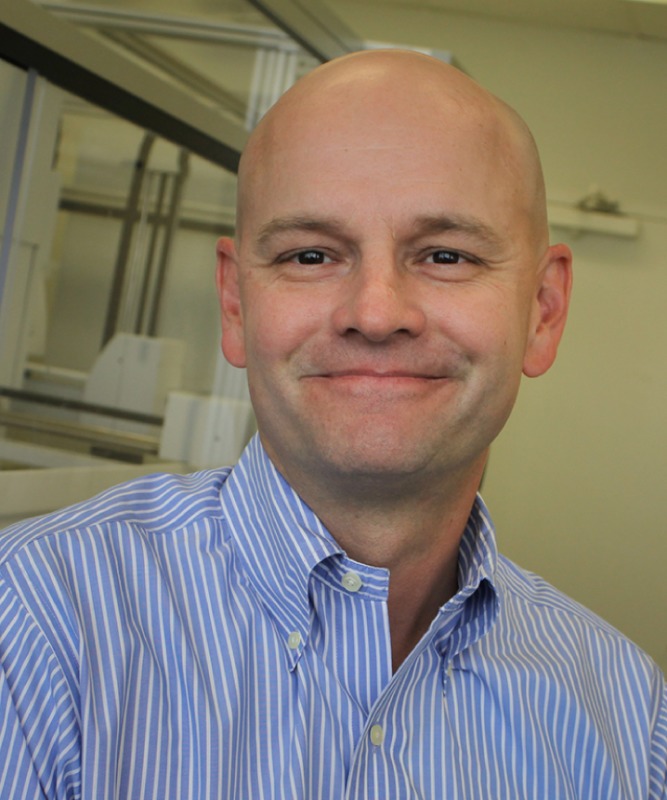


Geoffrey W. Smith was born in 1965. He obtained a Bachelor of Arts degree from Williams College in Williamstown, MA. After a stint as a Research Associate at Harvard Business School, he graduated from the University of Pennsylvania Law School. Following a federal court clerkship and first job experiences in law, he joined a healthcare services start-up named Advanced Health as one of its first employees. Geoff then co-founded various healthcare and technology companies, including Interbind and Ascent Biomedical Ventures, and is still a Managing Partner at the latter. In 2012, he joined the Icahn School of Medicine at Mount Sinai, first as Professor in the Department of Population Health Science And Policy, and then as Director and co-founder of the Design, Technology, and Entrepreneurship PhD program. Until December 2014, he was a Senior Editor at *Disease Models & Mechanisms*. Geoff is now Managing Director of Mars Ventures.

**Let's start with your background. You have a Bachelor of Arts degree and a law degree. How is it exactly that you ended up working in biotech and pharma?**

My career path has been anything other than linear. I was actually pursuing my legal career when two entrepreneurs turned up at the law firm I was working for with an idea for a new technology-based company focused on more effectively managing healthcare services. I was a new associate without much to do, so I got assigned to work with the start-up and after about a year they asked me to come and join the company, Advanced Health. I had grown up in a very medically oriented family – my father was a medical school professor, my older sister was a PhD, and my younger sister is a medical doctor – and so to a certain extent joining a start-up in the healthcare space was a bit like joining the family business.

We had a fair amount of success with that company. A little less than 2 years after I joined it, we had a successful initial public offering, and that started me down the road of participating in the start-up environment around healthcare.

**It sounds like it was not a surprising path for you. Which key people influenced you?**

Actually, it is somewhat surprising in that I had really prepared for and expected a career in law. Certainly, the work I did in law school and the first jobs I had after that started me on a different career. I was really focused on international relations and international law. The twist was that I got brought back into the healthcare arena, and ultimately the biotech arena, by a serendipitous connection – one of the entrepreneurs who started Advanced Health had trained at Brigham Women's Hospital where my father was the Chief of Cardiology. It was through this connection that I became more than just an associate drafting legal documents and really began to build a close relationship with the founders, which ultimately led to me joining that business. This taught me that you can spend much of your time preparing, and thinking that your schooling is going to take you in one direction, but individual relationships can change your path and take you somewhere else altogether. In my case, these particular relationships stemmed from my father, who clearly had an enormous influence on me. He was both a practising clinician and a basic researcher, studying basic biology related to the function of sodium and potassium in the heart, but he also did applied research. He helped develop a radioimmunoassay test to measure digitalis levels in the blood and ultimately was involved in developing a drug called Digibind, an antidote to digitalis toxicity, which was one of the first drugs to use antigen-binding fragments [Fab] as the basis for a drug. Watching him manage these different activities in his career had a big influence on me.

“This taught me that you can spend much of your time preparing, and thinking that your schooling is going to take you in one direction, but individual relationships can change your path and take you somewhere else altogether”

I think that each of my sisters – as I said, one of whom went down a PhD route and one of whom went down a medical training route – had a big influence on me, as well. Watching the challenges that they had to face in those areas in some ways pushed me to go off towards law school and take a different path. It also brought me back to one of the aspects that I think is the most rewarding in the bioscience field, which is that you can have a profound impact on a large number of people through your efforts, whether they be purely research-based, academic-based or commercially-based.

**One of the things I was constantly impressed by is that you always seem to have a good feel for the health field and the biology field. Is this because it is something of a family business?**

I think so. Growing up at my dinner table, I was just privileged to get to meet and interact with a lot of incredibly successful clinicians and researchers. For me those were comfortable conversations: these were friends and so there was a comfort level being involved in that environment. I didn't feel a lot of intimidation from it, which I think sometimes people who come from the outside do.

One of the aspects I really like about the bioscience field is the impact of ideas. Success is really about one's ideas and ability to execute them, and that was very appealing to me. It wasn't about how much money you had or where you went to school, it was really about the ability to think deeply about a problem or a potential advancement and figure out a way to find a way forward. It is also a very people-driven process because it is not only about thinking deeply yourself but also about thinking deeply with those in your field or adjacent to your field. Lots of different personality types can succeed in this field, but I think it is certainly easier for people who have an affinity for sitting with people and thinking about a common area of interest.

**To that point, you actually have walked between business and scientists. What do you see is the difference? Some of the priorities are obvious, but what are the differences in terms of what motivates people in the two? Are the personalities that you come across different between the basic science world, the translating science world and the business world?**

I don't think the personalities are particularly different. I think you find introverts and extroverts and everything in between in each of these areas. I am not sure that personality is necessarily a good predictor of success. I think it's a question of what toolset you are most comfortable using to get at a problem, and where in the lifespan of a problem you're interested in working.

For example, scientists in academia very often are interested in working at an early stage of a problem. They understand something fairly basic about a process or something earlier in the understanding of a field. People who gravitate towards industry, instead, are more excited about working on the later part of a process, so, rather than trying to understand what the fundamental working mechanism is, they want to understand how to work that mechanism in a way that is predictable and repeatable.

Obviously people in the commercial realm are often highly influenced by money, but even that I don't think is really particularly the differentiator. There are plenty of academics who are driven by money as well. I really think it has much more to do with where on the spectrum of understanding one is interested in working. Industry is geared to solving practical problems and, if a lot is understood about a problem, to getting down to the ability to repeatedly and safely intervene, whereas academia really lends itself more towards understanding the front end of a problem or of an unknown mechanism to understand it first and at a more basic level.

**What about working in teams versus individually? Do you see a difference there?**

I think that has changed over time. I think it is very hard in academia today to be the brilliant solo investigator. I'm not saying it's impossible but, considering the increasing size of the data sets one is working with, the statistical methods one has to use, the complexity of different fields overlapping with each other, it's just very hard to handle all the necessary aspects of modern science as an individual. Increasingly, working in teams isn't a choice: I think it's a necessity in order to be effective. The difference may be that, in academia, often the teams are teams of collaborators (meaning they have influence but not necessarily power over all people participating in the team) who may work for different institutions, whereas, more commonly in industry, teams are working within a single corporate structure. In industry more often there are hierarchical relationships, which may allow for more directive behavior. Again, I'm not sure I would draw as much distinction between team or not team and between industry and academia, but I might draw somewhat of a distinction between how those teams function and how one manages a team. I think they are a bit different between the two realms.

“Increasingly, working in teams isn't a choice: I think it's a necessity in order to be effective”

**Let's turn to *Disease Models & Mechanisms* [DMM], where you have been a Senior Editor. What did your experience at DMM teach you about science publishing that perhaps you hadn't thought about, and has it made you think more deeply about what goes into a good scientific piece of work? What were some of the surprises?**

Watching the detailed process that is necessary to take a piece from an initial submission through to a published article gave me comfort and respect for the level of diligence and the level of attention that the reviewers brought to the vast majority of the pieces. It gave me a good feeling that the science community can be a strong self-reinforcing organization that takes its responsibility to heart and only publishes the best of the work available. I think that was very reassuring.

An interesting question for me was: is there a different function that the publication process could play in helping to galvanize new ideas or new interactions among different fields? That seemed to be challenging because people don't want to rush out there without their ideas and data being fully thought-out and fully vetted. But still, somewhere in my mind is this notion that there should be an option in the publishing world to play a little bit earlier in the generation of new ideas.

**Do you mean journals having an earlier relationship – earlier in the experimental process with a laboratory – to work with them to provide advice?**

I don't know if it's to provide advice. One of the things I was struck by at DMM is that there are these different siloed research communities – for example, the fly and the fish communities – in which interactions and relationships in the individual fields are so well established and routinized. And the outcome from a publishing standpoint is still the canonical academic paper that has been relatively unchanged over a long period of time. Yet, we have had these tremendous changes in information and communication technology such that the manner of knowledge production and the methods of communicating in other parts of society have changed dramatically. It feels like there hasn't been nearly as big a concomitant communication change in the biomedical sciences, and so the silos and the standard paper remain the way things are done.

The publication process, because of its preciseness, can take quite a long time, so the musing here is whether there is a way that the publishing industry could facilitate an earlier, more speculative communication of interesting results in a way that would positively impact the field by turning over new information sooner. If you look at an area like maths, for example, and their pre-print servers, there is more of a notion of putting ideas out in the community that acts as a kind of peer-review process and a way to get the community interacting on new ideas early. That doesn't seem to get a lot of attention in the life sciences area. It seems to me that even journals like the *PLOS* journals that are pushing towards a more open world of communication are still ending up being pulled back into the canonical paper form to communicate.

“…the musing here is whether there is a way that the publishing industry could facilitate an earlier, more speculative communication of interesting results in a way that would positively impact the field by turning over new information sooner”

**I guess one of the issues on the biology side is that there is a real emphasis on trying to get your paper into the most prestigious journal, so people don't want to drop that paper until it is as far along as possible to aim at high-impact journals.**

That of course becomes a self-reinforcing system. If the yardstick used in the life sciences industry is publication in high-impact-rated journals, then you are going to get that behavior. But if you're interested in the generation of new knowledge and in moving your field forwards, it is at least plausible that publishing in a quicker fashion or with at least some outlet to move more creative ideas ahead would be attractive.

There are clearly challenges to that. But I do think it's remarkable that if you look at almost every other media area there has been a huge amount of change since the advent of the internet era, but there really has been very limited change around life sciences publishing. It's been surprisingly conservative to me. I am wishing there would be more experimentation to find other ways to communicate information sooner and in a way that could spur more creativity.

Of course, when you tie publishing back to industry, for competitive and intellectual property protection reasons, industry tends to not really want to get out in the front with its most interesting work too early. I think that a lot of things being published out of industry are not the most interesting stuff that is happening. But again it seems to me that another area that science publishing should be thinking about is how they could come up with other solutions that might provide for a more creative interaction between publishing and industry.

**Talking about old models versus new models, let's move to issues of training. Another area that you've been impressive at is the training of scientists. You've had your hand in creating a new PhD track at Mount Sinai called Design, Technology, and Entrepreneurship [DTE]. What is your view about how we train scientists, what we're doing better these days and what you would like to see being done better to train them?**

It seemed to me that there was a remarkably small amount of experimentation in academia around thinking about how to train biomedical PhDs, and that academia had missed the opportunity to provide a better set of tools to PhDs to allow them to be effective across a wider range of potential career outcomes. The majority of biomedical PhDs are not ending up in tenure-track faculty positions but rather in the ‘alternative career track’. It seemed to be disingenuous to train them solely for the academic track if in reality the majority were going to some other career track.

So what I was really excited about in putting together the DTE program was trying new ways to train PhDs to be effective askers of questions and proposers of solutions, and to create an environment where they could gain experience in how to solve a variety of problems effectively.

“…what I was really excited about in putting together the DTE program was trying new ways to train PhDs to be effective askers of questions and proposers of solutions, and to create an environment where they could gain experience in how to solve a variety of problems effectively”

This meant that our students had to be rigorously trained as scientists, but this was an ‘and’ opportunity and not an ‘or’ opportunity. In addition to being trained as excellent basics scientists, we wanted to give them some training in how engineers think about problems, how designers approach issues, what tools those people use and how that impacts how they try to solve a problem. Hopefully over time this would produce students that are better suited for interacting and influencing other parts of society – be it industry, government or policy – and better positioned to compete in what is a very competitive job market.

**What were some of the things you did in the DTE training to get at this?**

We really tried to teach theory in the context of real problems. Virtually all the classes of the DTE curriculum were problem-driven. We created a class that we called ‘The Q.E.D. Project’ that followed along from efforts at Stanford and elsewhere to teach students how to identify an unmet need. We then asked them to form a team to address the unmet need, and then helped them understand how to build a prototype to address that need. Along the way, we also talked about what kind of roles people in their team need to play. Should your team be very diverse or very deep in a given area? How do we integrate people who have different cultural backgrounds or how do we integrate medical students with PhD students? We brought in a lot of people out of the non-academic environment who were practitioners and experts in their various areas and we tried to get students to think about the full range of stakeholders they would have to engage with to bring a solution to bear.

We did not want to spend a lot of time lecturing the students in a purely didactic way. We wanted to engage them in a process where they were solving important problems as part of the class. Whether that was a class on modelling or an engineering-focused class, or how to think about scientific problems, the core of DTE was built around getting the students to grapple with a real world problem and let all the learning hang off that.

**How did the students respond to that? Do you think you were successful?**

Based on the number of students signing up to take the courses and the student evaluations after the classes were over, I think we really struck a chord. I wouldn't say it was necessarily the right answer for every student but I think there is clearly a group of students for whom this is a really effective and motivating approach.

**Let's now move to drug discovery and development – the focus of the new online Special Collection from DMM. What would you say are some of the most urgent challenges in drug development that you have seen?**

I think one of the most urgent challenges is to begin to break free of some of our ‘old’ ways of thinking and take advantage of new scientific insights. For example, if you look at the traditional organization in a medical school environment, they are centered around departments devoted to organs (liver, heart, kidney). I think our increasing scientific understanding is that there are disease processes that may impact multiple organ systems but ultimately it is understanding the process, and drugging the process, that becomes important and not drugging the organ.

I think that moving towards a process-oriented understanding of what common mechanisms are implicated in a given disease state or therapeutic challenge will help us be a little more creative and a little bit more interdisciplinary in how we think about these challenges.

**One of the difficulties with those new approaches is that pharmaceutical companies and academic institutions have not had a great track record of working together. Do you think that's true? And why do you think it's been so difficult to move ideas from the bench to the clinics?**

I think this is complicated. If you take the academic researchers' point of view, their early identification of a problem and early identification of a potential solution feels like they have moved the ball very far forward towards the end solution. If you take industry's point of view, the identification of the target or even the identification of a potential chemical compound is really just barely beginning to get to the starting line; the bulk of the time and the bulk of the dollars that will ultimately be needed to create a product come after the academic work and these will be spent by industry. I think that this differing point of view around where and how value is created has a lot to do with many of the challenges that arise when academia and industry are speaking to each other.

**Is it important to bridge this gap or is everybody playing their role?**

I think there's an opportunity for academia to continue in its current role but to carry the potential solution further. I think in certain areas the access to tools and to patients allows academics to maybe carry projects further and closer to ‘proof of concept’ than they did historically, and that will continue to add value to the academic institution. That would ultimately help to bridge this gap because if you've taken something closer to proof of concept while still within the academic institution, you have created more value, you are able to engage with industry differently, and maybe the value perception gap is closed somewhat.

What industry is really good at is organizing and managing late-stage research and clinical trials in an effective manner, and what academia is really good at is understanding basic questions, finding targets and sometimes finding early chemical compounds. Again, I think that the perception in academia of where value has been created is in part related to the fact that many academics haven't been given the exposure or the training to actually understand the full breadth of the drug-development process. While they may have a general sense of it – we have all seen the same diagrams showing the steps and the funnel narrowing down from a million compounds to something getting onto the market – only those with real exposure to the work in industry understand it at a visceral or experiential level. One of the opportunities for academia is to find better ways to have some cross-talk, whether that's internships for graduate students to get some experience in industry or other ways to get the students really exposed to the industrial side of drug development. Obviously, all the trained scientists on the industry side have been through academia because they had to go through it to get their PhDs, and thus they understand the academic side of the house pretty well. I really think the challenge is getting to people who have spent their whole career in academia to have a better understanding of what the drivers are on the industry side.

“One of the opportunities for academia is to find better ways to have some cross-talk, whether that's internships for graduate students to get some experience in industry or other ways to get the students really exposed to the industrial side of drug development”

**Between target identification and clinical trials of course there is another piece. At what point does the researcher in academia put down his pipette, walk out and start a biotech company? Should that happen?**

That's a fraught question because I think it is an enormous undertaking to start a biotechnology company. Fundraising, intellectual property, regulatory affairs, company management – there are a whole number of disciplines that biotech companies have to take on. It is very rare that an academic scientist is going to have the training, the time and the motivation to do all of those things while also continuing to pursue their academic career in a very challenging funding environment. I think it comes back to this point that we were talking about with teams. I think it is really important for a scientist who is excited about their work and thinks it may be the basis of a company to go out and begin to form a team that is going to increase the likelihood of success. They have to accept the fact that science is a critical component, but it is just a component, and many different disciplines along with many different people are needed to make a successful company. If a scientist can bring that sort of collaborative view point and is open to working closely with an intellectual property attorney, with a business development person and with whomever their funding source is, that will increase their likelihood of success. They have to do it with a certain amount of humility, which is to say that it isn't just going to be the science that drives the success: all the given pieces have to come together to be successful.

**You've watched a lot of technology coming through, including at your new position at Mars. Which technology excites you?**

We all have to pay a lot of attention to CRISPR and the gene-editing technologies. There is certainly a number of intellectual property issues that have to get sorted out but that's clearly an area that will have a huge impact not just on human health but on animal health and plant health as well.

The other area I've been thinking a lot about lately is the microbiome. As sequencing technology has altered in cost and time, we have begun to be able to explore the microbiome in a way that historically was not possible. And it feels like we are moving towards a tipping point where the explosion of understanding is going to open up a lot of interesting opportunities for us to intervene. Whether that's through traditional drug modalities or through altered nutrition or through changing the microbial community in soil to produce crops that have higher nutrient value or other approaches, I think that's another broad area that seems poised to begin to offer really interesting results.

**Were you surprised that a company like Mars, which has not been a basic research company at all, is now giving you an opportunity to build something that is much more research-orientated?**

The reality of Mars is that they have actually had a very deep fundamental research program for a number of years. They got involved in the sequencing of the cacao genome and contributed it to the public domain, and they are now also involved in the sequencing of the genomes of a large number of orphan crops in Africa. So they have been very active in their research both in the company and in collaboration with academic scientists around the world. The nature of the company has meant that the work is perhaps not as obvious as others, but it is a remarkably science-driven company in much of what it does.

**You have done a myriad of things. What are the one or two things that you are most proud of?**

I am most proud of my efforts to keep a hand in both the commercial and the academic world. It certainly has not been easy but I have received enormous satisfaction from the opportunity to work with bright students at each of the schools I have had the opportunity to teach at. I am not sure there is anything more satisfying than the opportunity to work with students and feel you have helped them towards their goals.

At the same time, I think I've been effective in doing that because I have managed to keep an active role in the applied world. In some ways, my greatest achievement has been finding a way to balance those two interests in a way that seems to have worked for the various organizations I've been affiliated with.

**How do you relax away from work? Do you have a family?**

I am married. My wife is a securities litigator so has a very active career of her own. We have two children, one in high school and one in middle school. I've had the privilege to coach both of them on their various soccer teams since they were each about 4 years old so that's been a lot of fun.

The other thing that many people will not find relaxing – but for some reason my family does – has been taking backcountry ski trips annually for a number of years. Worrying about navigating through the snow and finding shelter before darkness falls has a way of clearing the mind.

## Notes

This article is part of a subject collection on Model Systems in Drug Discovery: from Bench to Patient. See related articles in this collection at http://dmm.biologists.org/cgi/collection/drugdiscovery

